# Semantic Framework of Internet of Things for Smart Cities: Case Studies

**DOI:** 10.3390/s16091501

**Published:** 2016-09-14

**Authors:** Ningyu Zhang, Huajun Chen, Xi Chen, Jiaoyan Chen

**Affiliations:** Computer Science and Technology Institute, Zhejiang University, Hangzhou 310058, China; zhangningyu@zju.edu.cn (N.Z.); xichen@zju.edu.cn (X.C.); jiaoyanchen@zju.edu.cn (J.C.)

**Keywords:** Internet of Things, smart city, energy management, traffic pattern

## Abstract

In recent years, the advancement of sensor technology has led to the generation of heterogeneous Internet-of-Things (IoT) data by smart cities. Thus, the development and deployment of various aspects of IoT-based applications are necessary to mine the potential value of data to the benefit of people and their lives. However, the variety, volume, heterogeneity, and real-time nature of data obtained from smart cities pose considerable challenges. In this paper, we propose a semantic framework that integrates the IoT with machine learning for smart cities. The proposed framework retrieves and models urban data for certain kinds of IoT applications based on semantic and machine-learning technologies. Moreover, we propose two case studies: pollution detection from vehicles and traffic pattern detection. The experimental results show that our system is scalable and capable of accommodating a large number of urban regions with different types of IoT applications.

## 1. Introduction

The rapid development of Information and Communication Technologies (ICT) and the Internet of Things (IoT) has affected cities in the form of changes to the physical infrastructure, buildings, urban transportation systems, governance, healthcare, etc. The integration of devices, platforms, and applications using ICT is of great importance to smart cities. According to [1], approximately 50 billion devices are expected to be connected to the Internet, generating enormous amounts of data for applications in a variety of areas such as transport, e-health, energy management, and the environment. Data of such a widespread nature is potentially valuable and can improve our lives by revealing latent patterns through mining. However, exploitation of the senses to implement artificial intelligence necessitates unification of the IoT data by existing technologies. In fact, many efforts have been devoted to this purpose and various solutions have been proposed in this area. The W3C founded the Web of Things Community Group and initiated the standards oneM2M [2], which are aimed at developing the technical specification for a common IoT service layer by reusing existing web standards and protocols, including Restful, HTTP, and Resource Description Framework (RDF).

However, the IoT data obtained for smart cities are extremely complex, thereby posing a series of challenges. For example, POIs (Points of Interest) are represented by spatial points associated with a static category, whereas air quality is represented using geo-tagged time series. Human mobility data is represented by trajectories. Thus, different types of data have different forms of representation, distribution, scales, and densities, all of which results in the different data types existing in isolation. Moreover, there exist certain correlations between the different data in regions of smart cities. For instance, the function and POIs of a street block may indicate the concentration of traffic in a region during a certain time (e.g., rush hour), whereas the traffic volumes and speed of vehicles in the same block may indicate the air quality (PM2.5), and the low terrain value of the block may indicate the occurrence of waterlogging. Furthermore, different blocks in cities have different populations and infrastructure, which may lead to data sparsity for some regions. In fact, there exist many applications for different areas and aspects of smart cities based on machine-learning technologies. However, most of these applications focus on specific problems and do not have the necessary capability to interpret the data.

The technologies of the Semantic Web are viewed as a key element of the IoT. In fact, oneM2M offers a general model supporting the semantics for the IoT. An abstraction and semantics layer is provided to solve the interpretability of data by defining some standard concepts. Moreover, the Semantic Web offers an interface to facilitate the fusion of IoT data with existing knowledge such as Linked Data [3] and Wiki Data [4]. Currently, many studies aim to provide definitions and annotations of various WSNs by providing corresponding description ontology. However, a comparatively small amount of work focuses on the processing of IoT data from smart cities.

In this paper, we present a semantic framework for integrating the IoT with machine learning for smart city applications. In practice, we first divide the city into blocks. We analyze the types, sources, and structures of urban data and standardize the forms of data to fuse them across modalities. In fact, we divide the data from smart cities into three categories: (1) Background Knowledge, which is almost time-independent, such as POIs, road networks, terrains, function zones of a block; (2) Sensor Data, which is generated by sensors and published by various web services such as bus and taxi trajectories, air quality, and traffic; and (3) Social Data, which is generated by people such as on social media, in user comments, and so on. We can obtain each of the three kinds of data for a certain block from the web services of a smart city. For each block, it then becomes possible to learn the latent features by using fusion technologies to generate urban knowledge based on all three kinds of data according to application demands. We use semantic technologies to model and annotate all kinds of data to enhance the meaning of data values and hide the complexity of data sources and environments by providing a standard format to represent. Moreover, we adopt a utility layer to integrate machine-learning methods such as transfer learning for solving data sparsity. Finally, we include two case studies: pollution detection from vehicles and traffic pattern detection, by using data obtained from taxi trajectories and traffic based on our framework.

The major contributions of this paper are as follows:

(1) We present a semantic framework for the IoT integrated with machine learning for smart city applications.
(2) We build an application-specific urban knowledge graph and include two case studies involving pollution detection from vehicles and traffic pattern analysis and analyze their potential causes.
(3) We evaluate the practicability and scalability of our framework by implementing it on a SPARK cluster with two case studies.

The remainder of this paper is organized as follows. Section 2 briefly reviews existing studies on the semantic IoT and smart cities. Section 3 contains the framework, urban knowledge graph, typical technology, and use cases. In Section 4, we present the results of our experiments. Finally, Section 5 summarizes our findings and concludes the paper with a brief discussion on the scope for future work.

## 2. Related Work

### 2.1. Semantic IoT

To the best of our knowledge, our framework is the first involving semantics integrated with machine learning for IoT data of smart cities. The major task of the IoT is to represent the "things" by standard schemas. The W3C have developed an ontology [5] representing sensors and data, providing metadata for spatial, temporal, and other objects. However, their work mainly focuses on defining a standard ontology for annotation of the IoT. In addition, many semantic IoT applications have been proposed such as naturopathy applications based on multiple datasets [6]. Hu et al. [7] developed a SSEO, which is aimed at semantic indexing and event detection by way of machine processing. Other applications include Open-Multinet [8] and so on. Amelie et al. [9] proposed a a semantic engine applied to IoT and smart cities. Chen et al. [10] proposed large-scale real-time semantic processing framework for IoT. However, their work was mostly aimed at specific IoT applications rather than a generic IoT framework for smart cities. Moreover, the idea of a semantic framework integrated with machine learning for smart cities has not received much attention.

### 2.2. Smart City

Urban computing is a process of acquisition, integration, and analysis of a large amount of heterogeneous data generated by diverse sources in urban spaces, such as sensors, devices, vehicles, buildings, and humans, with the aim of addressing the major issues cities face (e.g., air pollution, increased energy consumption, and traffic congestion) [11]. According to [11], there are three main challenges associated with urban computing: urban sensing and data acquisition, computing with heterogeneous data, and hybrid systems blending the physical and virtual worlds. In recent years, many applications have been proposed in different scenarios of smart cities including transportation [12], the environment [13], energy [14], social [15], the economy [16], and public safety and security [17,18]. Moreover, the Intelligent Transportation Systems (ITS) have rapidly developed in Europe. Most of their work constructed features for specific domains and adopted machine-learning methods. However, this work did not include semantic interpretation of the results and focused on specific domains. In general, data obtained from smart cities are usually not such that they can easily be understood by humans. For example, the air quality recorded by sensors is usually represented by values such as “53” (PM2.5). These representations would be more meaningful if we had the semantic meaning of the numerical values such as “slight pollution”. Moreover, we can explain the results of some predictions by using common rules.

## 3. Approach

### 3.1. Framework

**Figure 1:**
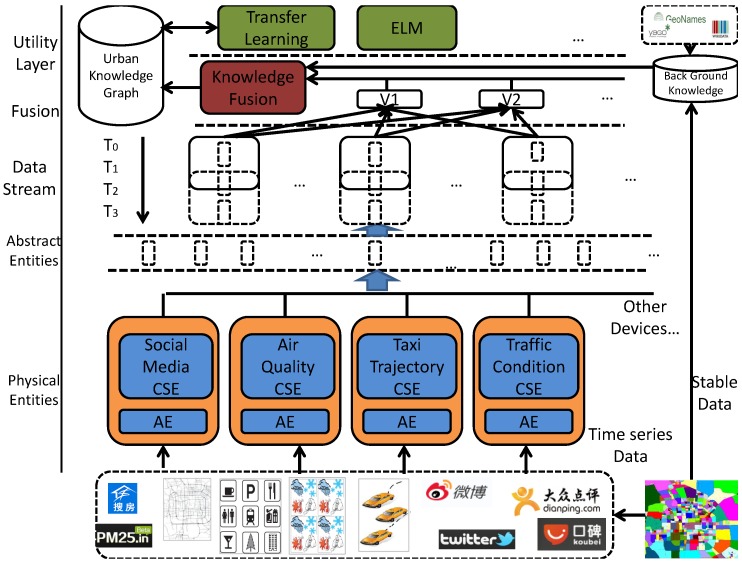
Semantic framework of IoT integrated with machine learning for smart city applications.

Figure 1 shows the architecture of our semantic framework of IoT integrated with machine learning for smart cities. It consists of five parts: (1) Utility layer; (2) Fusion layer; (3) Data stream layer; (4) Abstract entities layer and; (5) Physical entity layer. The data is transmitted from the bottom to the top of the framework. Each layer has its necessity of existence.

### 3.2. Urban Knowledge Graph

We firstly divide the smart city into blocks according to the road networks. The different colors in the rectangle in the lower right corner of Figure 1 represent different blocks. For each block, we obtain amounts of stable (time-independent) data from OpenStreetMap [19] and the APIs of Google [20] and Baidu [21], including POIs, the terrain, and road networks. We also map the locations of blocks with entities from Yago2 [22], Geoname [23], and WikiData [24] to enrich our knowledge of a smart city. For instance, we may obtain a POI category “coffee shop” for “Starbucks” in a block. We can enhance the semantic meanings by matching the entities with the external knowledge bases. In this way, we may be able to obtain additional knowledge about “Starbucks” and “coffee shop”, e.g., “where are the closest coffee shops”, or “where is the largest Starbucks outlet in the city”.

Then, physical entities collect raw data in real-time from social media and physical sensors. Each kind of sensor is to be organized according to its logical entity (AE) and common services entity (CSE), providing common and logic services for applications. Afterwards, the data is received by the abstract entities layer to add semantic annotations. This layer hides the complexity of devices by providing a standard format to represent data from all kinds of devices. Thus, it now becomes possible to view complex raw data as unified data streams. The data-stream layer extracts the data streams into windows according to the requirements of the upper applications. The RDF streams in the data-stream layer are a quad (<s,p,o,t>), which is defined as ordered pairs constituted of multiple data units. For example, the data stream is denoted $S_{i}=\left\{B_{i}^{0},B_{i}^{1},..B_{i}^{j}\right\}$, where *j* is the timestamp and *i* is the id of the sensor. The window is a subset of RDF streams for a given time range. For real applications such as traffic monitoring, disaster management, and environmental monitoring, the data take the form of continuous streams.

The fusion layer is the key to our framework. This layer has three main tasks: (1) receiving external requests and creating the corresponding virtual entities; (2) knowledge fusion with data from the background knowledge base, producing continuous results from both social data and sensor data; and (3) converting these data into an urban knowledge graph. In practice, each virtual entity is aggregated by the RDF streams from several corresponding windows and auxiliary background knowledge from external knowledge bases. For example, if a user requests the air quality of a particular street block, a new entity is generated as a result of the aggregation of data from related sensors and social media (e.g., PM2.5, AQI, tweets from the department responsible for the environment). We then obtain the air quality from the newly generated virtual entity. The procedure of knowledge fusion generates latent feature vectors for each block through background knowledge, social data, and sensor data according to specific application demands. For instance, if the user requires a forecast of the traffic conditions $y_{i}\in \left\{0\left(Clear\right),1\left(SlowSpeed\right),2\left(Jam\right)\right\}$ of street block *i*, the traffic-related information (e.g., weather, historical traffic situation, and time ) is fused into feature vectors through urban knowledge fusion, and this is used to train a model to predict result $y_{i_{1}}$. The technical details are discussed in the next section.

**Figure 2:**
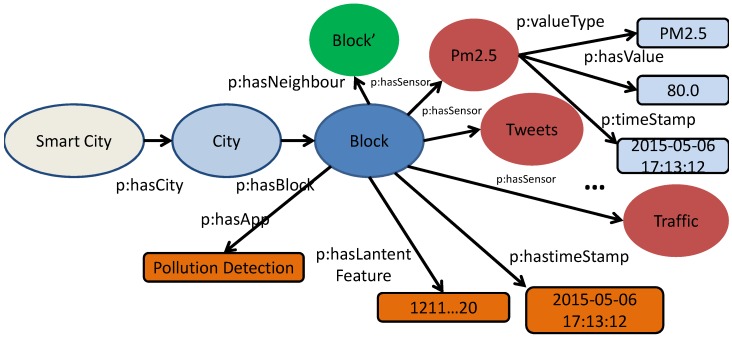
Simple concept model of urban knowledge graph.

We build the urban knowledge graph based on background knowledge data and the data that is collected during a subsequent period of time from smart cities. Figure 2 shows a simple concept model of an urban knowledge graph (the size of the figure is limited to enable more resources of a block to be shown). The model captures both types of resources for each block (PM2.5, AQI, traffic, terrain, weather, POIs) and the latent features (represented by a vector and a timestamp) through knowledge fusion, where “p” is the namespace of the properties. Each sensor has three properties: type, value, and time.

The urban knowledge graph offers three main advantages: (1) the linkage of different types of urban data indicates the potential condition of smart cities, which facilitates acquisition of related features by the real application. Considering traffic prediction as an example, we may have to find all the features related to a particular block that have an effect on the traffic, but may omit some influential factors such as those relating to the immediate vicinity. With the urban knowledge graph, all the nodes linked with blocks have potential effects for specific applications; (2) the knowledge graph may help to explain the results produced by the machine-leaning method to improve our understanding of smart cities. For example, the results indicate that one of the blocks experiences a traffic jam every day at 20:00 (i.e., outside of the rush hour). Although this seems inexplicable while using the urban knowledge graph, we find that the particular block is located near a train station at which many trains arrive daily at 20:00; and (3) the integration of machine learning and semantic knowledge is complementary in terms of the advantages and disadvantages of each of these sources of information. For example, a certain block with a low population and poor infrastructure presents a data sparsity problem, which is problematic for semantic technologies. In this regard, machine learning enables us to transfer knowledge from blocks with adequate data to build a robust model from which to infer results.

### 3.3. Typical Technologies

#### 3.3.1. Data Preprocessing for Smart Cities

**Figure 3:**
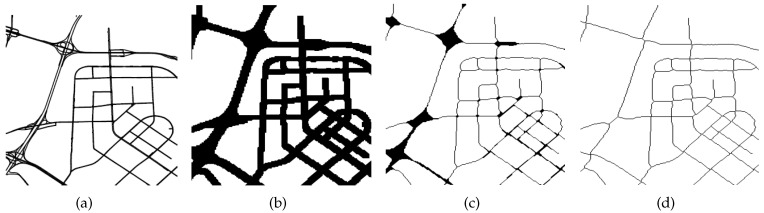
Procedure of segment maps. (**a**) Source binary image; (**b**) Binary image after dilation; (**c**) Binary image after thinning; (**d**) Final segmented regions.

We divide a city into disjointed blocks, assuming that placement in a block *g* is uniform. The road network is usually composed of a number of major roads, such as the ring road, whereas the city is divided into areas. We map the projection of the vector-based road network onto a plane. Then, the road network is converted into a raster model by gridding the projected map [15]. Actually, each pixel of the image of the projected map can be viewed as a block element of the corresponding raster map. Consequently, the road network is converted into a binary image (1 means road segments and 0 means blank areas) as Figure 3a shows. Then, we extract the skeleton of the road, while retaining the original two-value image topology through the iterations of dilation and thinning as the Figure 3b,c depict. Then, we find the connected 0 pixels (the blank area) in the binary image by classical algorithm introduced in [25], as Figure 3d shows. Finally, we obtain the blocks *g* of cities.

#### 3.3.2. Knowledge Fusion

For each block of smart cities, knowledge fusion is aimed at aggregating multiple disperse resources and learning latent features according to application demands.

We aggregate the urban knowledge by formalizing the task via concept filtering and recombination. Given data stream $S=\{S_{0},S_{1},...S_{n}\}$, the filter function is *γ* : S -> T, which is aimed at mapping RDF streams to triple set *T*, and the recombination mapping is *δ* : T -> T${}^{\prime }$. For example, in the case of traffic monitoring, *T* represents the triples containing the concentration of related sensors. *δ* denotes the mapping formula for computing the value representing the real traffic conditions (Jam, SlowSpeed, Clear). In addition, we adopt a reasoning mechanism to enrich the urban knowledge graph with implicit knowledge from semantically annotated data. For example, the PM2.5 value representing the air quality of each block is actually a number. We apply reasoning rules to derive new facts such as (Block${}_{id}$, Has Air Qualty, Good) if the value of PM2.5 is smaller than "30". The task can be formalized as $\left(S,B\right)\overset{\gamma }{\to }F$, where *S* and *B* are the data stream and background knowledge, *γ* is the set of rules, and *F* is the new facts.

**Figure 4:**
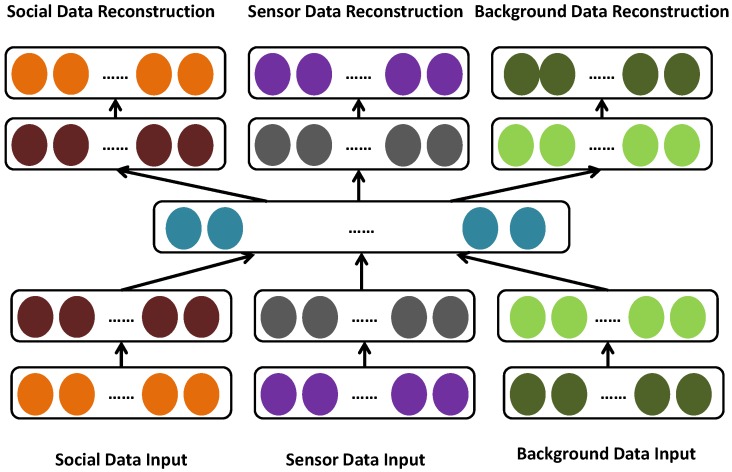
Urban knowledge fusion of learning latent representation.

We learn the latent features of each block by obtaining the social data, sensor data, and background knowledge data separately. According to previous studies [26], treating the features extracted from different data sources equally does not achieve the best performance. In fact, each kind of data has a different representation, distribution, scale, and density. In practice, each kind of data is represented as a set of feature vectors. Social data is composed of social media text, user comment texts, user ratios, and so on, according to the different application requirements. Sensor data is composed of values recorded by physical sensors such as the flow of taxis and buses, traffic congestion index, real estate, air quality, meteorological elements, and so on. The texts in social data are converted to vectors via a word-embedding procedure from GloVe [27]. We adopt the deep autoencoder [28] to capture the "middle-level" feature representation from these data. As depicted in Figure 4, the deep auto-encoder effectively learns (1) a more effective single modality representation with the help of other modalities and (2) shared representations by capturing the correlations across multiple modalities.

#### 3.3.3. Transfer Learning

The task of transfer learning involves transferring knowledge from rich data regions to regions with sparse data. For example, because of the large population and perfect infrastructure, social media data in large cities are relatively easy to obtain. However, less-developed areas or regions with fewer people have smaller populations and, hence, comparatively inactive social media. Therefore, it would be difficult to build a smart city system based on such data. To this end, we adopt transfer-learning technologies to enrich the feature representation for regions experiencing the data sparsity problem. In fact, the data in different regions have different distributions in terms of features. We adopt existing consensus-regularized auto-encoders of transfer learning [29] to construct a feature mapping from an original instance to a hidden representation, and we use the source domain data jointly to train a classifier for predictions on the target domain.

### 3.4. Use Case

#### 3.4.1. Pollution Detection from Vehicles

Pollution from vehicles is mainly effected by the volume and speed of traffic. We mainly calculate the PM2.5 value and the average value for a fixed time period in this case. For each block, we estimate the real traffic speed and volume of taxis, which have wide coverage of the city, and obtain the speed of each taxi and the total number of taxis (*n*). We adopt the average speed (*v*) of all sensors (i.e., taxis) as the traffic speed of this block. There are three reasons for that: (1) the taxis normally are driven randomly and have an average coverage in the whole city. Moreover, for a certain taxi (id), it is driven at almost any time in a day. However, for a private car, it normally has fixed routes and cannot appear on the road all the time; (2) there has been lots of research about using taxi data to estimate traffic volume such as [14,30,31] and so on; and (3) the taxi data are easy to obtain while data of private cars are difficult. We adopt *ϕ* times the number of sensors as the total volume of the block:

$$N_{i}=\phi \times n,\text{ where }\phi \text{ is calculated by }\phi =\left\{\begin{array}{ccc} \frac{V}{T} & if & \;\;block\notin Buz\\ 137 & if & \;\;block\in Buz \end{array}\right.$$

for Hangzhou, where *T* is the number of taxis in the city, *V* is the total number of vehicles in the city, and Buz is the set of business areas and regions with a large population mobility, such as a train station. The number 137 is calculated by using the static information of these kinds of regions in Hangzhou. In fact, there exist different environmental models to quantify the relationship between emissions and speed, such as MOBILE and COPERT. In this paper, we adopt the COPERT model [32] because vehicles in Hangzhou currently adopt European-3 standards. In practice, traffic emissions consist of hot emissions, cold start emissions, and evaporative emissions. The latter two types are omitted from our estimation due to the scarcity of data; in fact, they are also of less importance in terms of the overall emissions [33]. The hot emission factor (EF) is calculated by: $EF=\frac{a+cv+ev^{2}}{1+bv+dv^{2}}$. The parameters a, b, c, d, and e are given in the experimental section and are applied for Euro 3. The emission factors of PM2.5 are proportional to EF. For instance, the conversion factor for PM2.5 is $3\times 10^{-5}$. Finally, the overall emission for a certain block *i* is:

$$E_{i}=3\times 10^{-5}\times EF_{i}\times N_{i}\times L_{i},$$

where $EF_{i}$ is the hot emission factor of block *i*, $N_{i}$ is the volume of block *i*, and $L_{i}$ is the total length of the road in block *i*.

#### 3.4.2. Traffic Pattern Analysis

In this section, we discuss the mining of interesting traffic patterns. The problem is defined as finding the blocks that have vehicles flowing in or out during certain time periods and finding the reasons, which may be useful for the traffic control department and driver navigation [34]. In fact, this problem is mainly affected by the volume of traffic. For a block *g*, we try to use TAXI as the set of taxi trajectories of a city, each of which is denoted by a tuple <p,d>, where p is a pickup stop and d is a drop-off stop. We extract the arriving, departing, and transition volumes of taxis. Formally,

$$\begin{array}{c} F_{taxi}^{av}=\left|<p,d>\in TAXI:p\notin g\&d\in g\right|,\\ F_{taxi}^{lv}=\left|<p,d>\in TAXI:p\in g\&d\notin g\right|,\\ F_{taxi}^{tv}=\left|<p,d>\in TAXI:p\in g\&d\in g\right|. \end{array}$$

These features and *ϕ* (last section) enable us to calculate the traffic volume entering and leaving each block. We use the linkages of the urban knowledge graph to identify the potential reasons for this phenomenon.

## 4. Experiments

### 4.1. Datasets

All of our experimental data are obtained from the open web services listed in Table 1. We obtain free-text descriptions of places by adopting geoparsing [35] to convert text into unambiguous geographic identifiers (lat-lon coordinates). The experimental setup was a SPARK cluster comprising four machines, with each node consisting of an 8-core Intel Xeon CPU at 2.13 GHz with 32 GB memory (Hangzhou, China). All the nodes are implemented with CentOS6.4 with JDK-1.7.0, SPARK-0.9.0, Scala-1.1.0.1. We used Discretized Stream (DStream) to model the window streams. The operator provided by the SPARK like "map" is semantically translated into an aggregate of the urban knowledge. We adopt the deep auto-encoder [28] to implement urban knowledge fusion and create latent representation of blocks according to different applications. For blocks with sparse data, we adopt consensus regularized auto-encoders of transfer learning [29] to transfer knowledge from blocks with rich data. We set a=217, $b=9.6\times 10^{-2}$, c=0.253, d=4.21, e=0.65 for pollution detection from vehicles. We evaluate our experiment by using the PM2.5 values from stations as ground truth values. We adopt the root mean square error (RMSE) defined as $RMSE=\sqrt{\frac{\sum_{1}^{n}{\left(y_{i}-\hat{y_{i}}\right)}^{2}}{n}}$, where $\hat{y_{i}}$ is a prediction and $y_{i}$ is the ground truth.

### 4.2. Case Studies

#### 4.2.1. Pollution Detection from Vehicles

We used our framework to detect the pollution from vehicles in Hangzhou. As seen from the results in Table 2, the time periods from 8:00–9:00 and 17:00–18:00 show the best performance. Moreover, the values for the time period 8:00–9:00 gives almost the same as another time period 17:00–18:00 across all districts. The urban knowledge graph indicated it to be rush hour during these periods. In fact, lots of one-way streets exist in Hangzhou, which result in the very low speed of cars in rush hours and indirectly create more records that go through one block in the same time interval. The more taxi records in the same time interval, the higher the accuracy. However, all values across districts, Column 2 (11:00–12:00) differs almost by one compared to Column 1 (8:00–9:00) and Column 3 (17:00–18:00). This is mainly because of the relatively few data. We collected our experiment data in the summer while the weather is extremely hot. Few people take taxis in this time period, which results in the relatively low accuracy. We additionally found differences among the different districts of the city (Gongshu, Shangcheng, Xiacheng, Xihu, and Binjiang). The Shangcheng and Xiacheng districts are mainly located in the Central Business District (CBD); thus, they contain larger numbers of sensors (taxis) or records. Actually, the other three districts are spread across larger areas and have more blocks. However, they have smaller populations, and, consequently, they generate less sensor data (fewer taxis).

#### 4.2.2. Traffic Pattern Analysis

**Figure 5:**
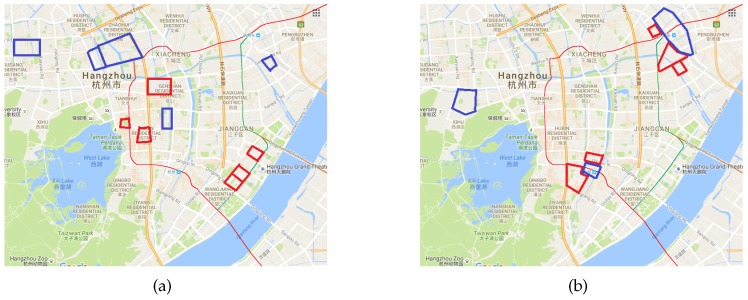
Traffic pattern analysis in Hangzhou. (**a**) Traffic pattern at 9:00; (**b**) Traffic pattern at 20:00.

We used our framework to mine the traffic patterns in Hangzhou. As Figure 5 shows, we identified interesting blocks that may indicate the potential traffic patterns in Hangzhou at 9:00 and 20:00. The areas outlined in red indicate blocks with considerable traffic inflow, whereas the areas outlined in blue indicate outflows. Actually, the red outlines in the image on the left recorded at 9:00 are located in the CBD according to the urban knowledge graph. Many people enter this area when driving to work, hence a considerable traffic inflow. The areas outlined in blue in the image on the left are mainly residential districts. However, the red areas in the image on the right (recorded at 20:00) are slightly unusual. We obtain the reason from the urban knowledge base and determine that these blocks have a commonality: all of them have a train station located in their immediate vicinity. At approximately 19:40, about 20 trains arrived in Hangzhou and this resulted in large flows of human traffic. This mined information would be useful for the traffic control department and for drivers for navigation purposes.

#### 4.2.3. Scalability

**Figure 6:**
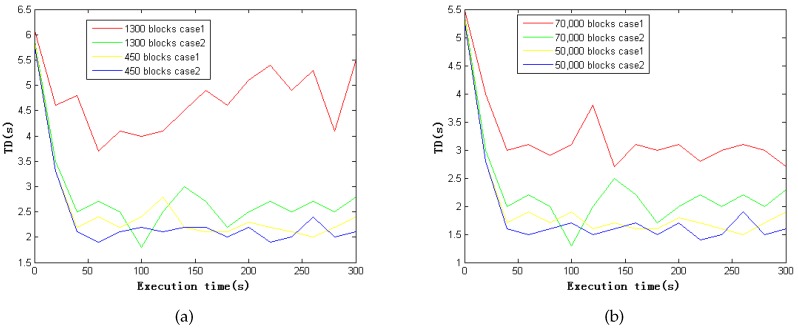
Processing time total delay(TD) with increased number of blocks. (**a**) TD with blocks in single node; (**b**) TD with blocks in cluster.

We used the two case studies to illustrate the scalability of our framework by increasing the number of blocks. We write the total delay (TD) into the log file after the data has been processed completely. In this experiment, we set the time slice (D) to 5 s and ran the program for 300 s. Figure 6 shows the processing time for a single node and for a cluster with different blocks. Case 1 refers to the case study involving pollution detection from vehicles, whereas Case 2 refers to the case study involving traffic pattern analysis. The number of blocks is varied from 450 to 1300. We performed the cluster experiment by duplicating the blocks using 50,000 to 70,000 nodes. For all figures, TD eventually stabilizes. The TD in the cluster is lower than for a single node, which shows that our system achieves excellent scalability.

## 5. Conclusions

In this study, we proposed a semantic framework to integrate the IoT with machine-learning techniques from the perspective of a smart city. We discussed two case studies based on the implementation of our framework and obtained interesting results. In addition, we tested our proposed approach for scalability. The results showed that our approach is applicable in practice and that it is highly efficient.

There are some limitations to this study, which should be addressed in future work. One major limitation lies in the partially missing data from some blocks and the limited availability of open data. For example, some data streams exist that do not have records when malfunction of sensors occur (a missing data stream in a time interval). We would like to mine the data of blocks more deeply in the future to estimate the missing data. The adaptability of this approach to real-world circumstances will also be considered in our future work. First, some visual analytics functions will be added to our ongoing demonstration system. Through presenting similar historical circumstances or forecasting results according to different features, the system will be able to provide more information for flexible decision-making. We are also investigating a new model that utilizes data from similar historical circumstances through understanding the underlying semantics of the data. We plan to apply our approach to additional applications. Moreover, we aim to study the distribution of our framework to enable it to process very large amounts of data.

## Figures and Tables

**Table 1:** Details of the datasets.

Datasets	Size (M)	Sources
Comments	2523	http://dianping.com
Tweets	11,023	weibo, twitter
Buses	254	http://chelaile.net.cn
Traffic	119	http://nitrafficindex.com
Real-Estate	35	http://soufun.com
Air	534	http://PM25.in
POI, Business Areas	10	http://map.baidu.com
Road Network, Terrain	9	http://openstreetmap.org
Meteorological	98	http://forecast.io,http://noaa.gov

**Table 2:** Overall performance of pollution detection from vehicles.

Districts	8:00–9:00	11:00–12:00	17:00–18:00
Gongshu	2.043	3.043	2.025
Xihu	2.712	3.689	2.712
Xiacheng	1.576	2.611	1.691
Shangcheng	1.783	2.691	1.721
Binjiang	2.581	3.511	2.522

## Supplementary Materials

The code of data preprocessing for smart cities are available at http://github.com/zxlzr/Segment-Maps, parts of our datasets are available at http://openkg.cn/dataset.

## Abbreviations

oneM2M	Standards for machine to machine and the Internet of Things
PM2.5	Air pollutants with a diameter of 2.5 micrometers or less
SPARK	A fast and general engine for large-scale data processing
W3C	World Wide Web Consortium
SSEO	Smart space event ontology
APIs	Application programming interfaces
IoT	Internet of Things
AQI	Air quality index
JDK	Java development kit
POI	Point of interest
COPERT	Model of air pollutant emissions calculation from road transport in Europe
MOBILE	Model of air pollutant emissions calculation from road transport in the USA
